# Cholesterol and Triglyceride Concentrations, COVID-19 Severity, and Mortality: A Systematic Review and Meta-Analysis With Meta-Regression

**DOI:** 10.3389/fpubh.2021.705916

**Published:** 2021-08-18

**Authors:** Angelo Zinellu, Panagiotis Paliogiannis, Alessandro G. Fois, Paolo Solidoro, Ciriaco Carru, Arduino A. Mangoni

**Affiliations:** ^1^Department of Biomedical Sciences, University of Sassari, Sassari, Italy; ^2^Department of Medical, Surgical and Experimental Sciences, University of Sassari, Sassari, Italy; ^3^Quality Control Unit, University Hospital of Sassari (Azienda Ospedaliero-Universitaria di Sassari), Sassari, Italy; ^4^Division of Respiratory Medicine, Cardiovascular and Thoracic Department, AOU Città Della Salute e della Scienza, Torino, Italy; ^5^Medical Sciences Department, University of Turin, Torino, Italy; ^6^Discipline of Clinical Pharmacology, College of Medicine and Public Health, Flinders University Adelaide, Adelaide, SA, Australia; ^7^Department of Clinical Pharmacology, Southern Adelaide Local Health Network, Flinders Medical Centre, Adelaide, SA, Australia

**Keywords:** cholesterol, triglycerides, COVID-19, disease severity, mortality

## Abstract

Lipid profile alterations have been observed in patients with coronavirus disease 2019 (COVID-19) in relation to disease severity and mortality. We conducted a systematic review and meta-analysis with meta-regression of studies reporting total, HDL, and LDL-cholesterol, and triglyceride concentrations in hospitalized patients with COVID-19. We searched PubMed, Web of Science and Scopus, between January 2020 and January 2021, for studies describing lipid concentrations, COVID-19 severity, and survival status (PROSPERO registration number: CRD42021253401). Twenty-two studies in 10,122 COVID-19 patients were included in the meta-analysis. Pooled results showed that hospitalized patients with severe disease or non-survivor status had significantly lower total cholesterol (standardized mean difference, SMD = −0.29, 95% CI −0.41 to −0.16, *p* < 0.001), LDL-cholesterol (SMD = −0.30, 95% CI −0.41 to −0.18, *p* < 0.001), and HDL-cholesterol (SMD = −0.44, 95% CI −0.62 to −0.26, *p* < 0.001), but not triglyceride (SMD = 0.04, 95% CI −0.10 to −0.19, *p* = 0.57), concentrations compared to patients with milder disease or survivor status during follow up. Between-study heterogeneity was large-to-extreme. In sensitivity analysis, the effect size of different lipid fractions was not affected when each study was in turn removed. The Begg's and Egger's *t*-tests did not show evidence of publication bias, except for studies investigating LDL-cholesterol. In meta-regression, significant associations were observed between the SMD of LDL-cholesterol and age and hypertension, and between the SMD of triglycerides and study endpoint and aspartate aminotransferase. In our systematic review and meta-analysis, lower total, HDL, and LDL-cholesterol, but not triglyceride, concentrations were significantly associated with COVID-19 severity and mortality. Cholesterol concentrations might be useful, in combination with other clinical and demographic variables, for risk stratification and monitoring in this group.

**Systematic Review Registration:** PROSPERO registration number: CRD42021253401.

## Introduction

Since the start of the coronavirus disease 2019 (COVID-19) pandemic significant advances have been made in the identification of specific patient characteristics that are associated with different disease severity and clinical outcomes. For example, pre-existing comorbidities such as hypertension, obesity and diabetes, clinical parameters such as hypoxia and CT-chest imaging findings, and circulating markers of inflammation, nutrition, hemostasis, and single organ function, have been investigated in observational studies in COVID-19 patients in order to develop and validate scoring tools for risk stratification and monitoring (1, 2). However, the continuing pressures on health care systems and the unpredictable progression of the pandemic, with new variants of the causative agent, severe acute respiratory syndrome coronavirus 2 (SARS-CoV-2), being discovered, additional efforts are required to further improve the performance of existing predictive tools (3). There is increasing evidence of significant alterations in lipid profile, particularly total cholesterol, LDL-cholesterol, and HDL-cholesterol concentrations in hospitalized patients with COVID-19. While increasing concentrations of cholesterol in the cell membrane have been reported to increase the binding activity of SARS-CoV-2, facilitating membrane fusion and the successful entry of the virus to the host (4, 5), clinical studies have shown acute reductions in plasma/serum concentrations of total cholesterol, LDL-cholesterol, and HDL-cholesterol in patients with COVID-19. Notably, the magnitude of this reduction seems to be proportional to the severity of the disease and might therefore assist with early risk stratification and clinical decisions (5, 6). By contrast, studies investigating the concentration of triglycerides in COVID-19 patients with different severity have reported variable results (6, 7). In order to capture and interpret the available evidence regarding the relationship between COVID-19 and lipid profile, we conducted a systematic review and meta-analysis of studies reporting plasma/serum concentrations of total cholesterol, LDL-cholesterol, HDL-cholesterol and triglycerides in hospitalized COVID-19 patients with different disease severity and clinical outcomes, particularly survival status during follow up. We hypothesized that COVID-19 patients with severe disease and/or not surviving during follow-up had lower plasma/serum concentrations of total cholesterol, LDL-cholesterol, and HDL-cholesterol, but not triglyceride, concentrations, when compared to patients with mild disease or favorable outcomes. A meta-regression analysis was also conducted to identify associations between the between-group total cholesterol, LDL-cholesterol, HDL-cholesterol, and triglyceride effect size and pre-defined biologically and clinically plausible variables.

## Materials and Methods

### Search Strategy, Eligibility Criteria, and Study Selection

A systematic literature search was conducted in the electronic databases PubMed, Web of Science and Scopus, from January 2020 to January 2021, using the following terms and their combination: “cholesterol” or “LDL” or “low-density lipoprotein” or “HDL” or “high-density lipoprotein” or “triglycerides” and “COVID-19” or “Coronavirus disease-2019” (PROSPERO registration number: CRD42021253401). Abstracts were screened independently by two investigators. If relevant, the full text of the articles were independently reviewed. The references of the retrieved articles were also reviewed to identify additional studies. Eligibility criteria included (i) assessment of total cholesterol and/or LDL-cholesterol and/or HDL-cholesterol and/or triglyceride concentrations in COVID-19 patients; (ii) investigation of COVID-19 patients with different disease severity, based on current clinical guidelines or admission to the Intensive Care Unit (ICU), or survival status, (iii) adult patients, (iv) ≥10 subjects, (v) English language, and (vi) full-text available. The references of the retrieved articles and reviews were also searched to identify additional studies. Any disagreement between the reviewers was resolved by a third investigator. We used the Newcastle-Ottawa Scale (NOS) to assess study quality, with a score ≥6 indicating high quality (8).

### Statistical Analysis

Standardized mean differences (SMD) were used to build forest plots of continuous data and to evaluate differences in total cholesterol, LDL-cholesterol, HDL-cholesterol, and triglyceride concentrations between COVID-19 patients with low vs. high severity or survivor vs. non-survivor status. When necessary, the mean and standard deviation values were extrapolated from the median and IQR values, as previously reported (9). The Q-statistic was used to assess the heterogeneity of the SMD across studies (the significance level was set at *p* < 0.10). Inconsistency across studies was evaluated using the *I*^2^ statistic where *I*^2^ <25% indicated no heterogeneity, *I*^2^ between 25 and 50% moderate heterogeneity, *I*^2^ between 50 and 75% large heterogeneity, and *I*^2^ > 75% extreme heterogeneity) (10, 11). A random-effects model was used, in presence of significant heterogeneity, to calculate the pooled SMD and the corresponding 95% confidence intervals (CIs). We also conducted sensitivity analyses to evaluate the influence of each individual study on the overall effect size with the leave-one-out method (12). The presence of publication bias was assessed by means of the Begg's adjusted rank correlation *t*-test and the Egger's regression asymmetry *t*-test at the *p* < 0.05 level of significance (13, 14). We also performed the Duval and Tweedie “trim and fill” procedure to further test and correct the possible effect of publication bias (15). This method recalculates a pooled SMD by extrapolating and incorporating the hypothetical missing studies, to increase the observed data so that the funnel plot is more symmetric. To explore possible contributors to the between-study variance, we investigated in meta-regression analysis the associations between the SMD and the following parameters: age, gender, specific endpoints (severity, ICU admission, or surviving status), study design (retrospective or prospective), white blood cell count (WBC), C-reactive protein (CRP), aspartate aminotransferase (AST), alanine aminotransferase (ALT), albumin, D-dimer, creatinine, diabetes, hypertension, and cardiovascular disease. Statistical analyses were performed using Stata 14 (STATA Corp., College Station, TX, USA). Our study was fully compliant with the PRISMA statement regarding the reporting of systematic reviews and meta-analyses (16).

## Results

### Systematic Research

**Figure 1:**
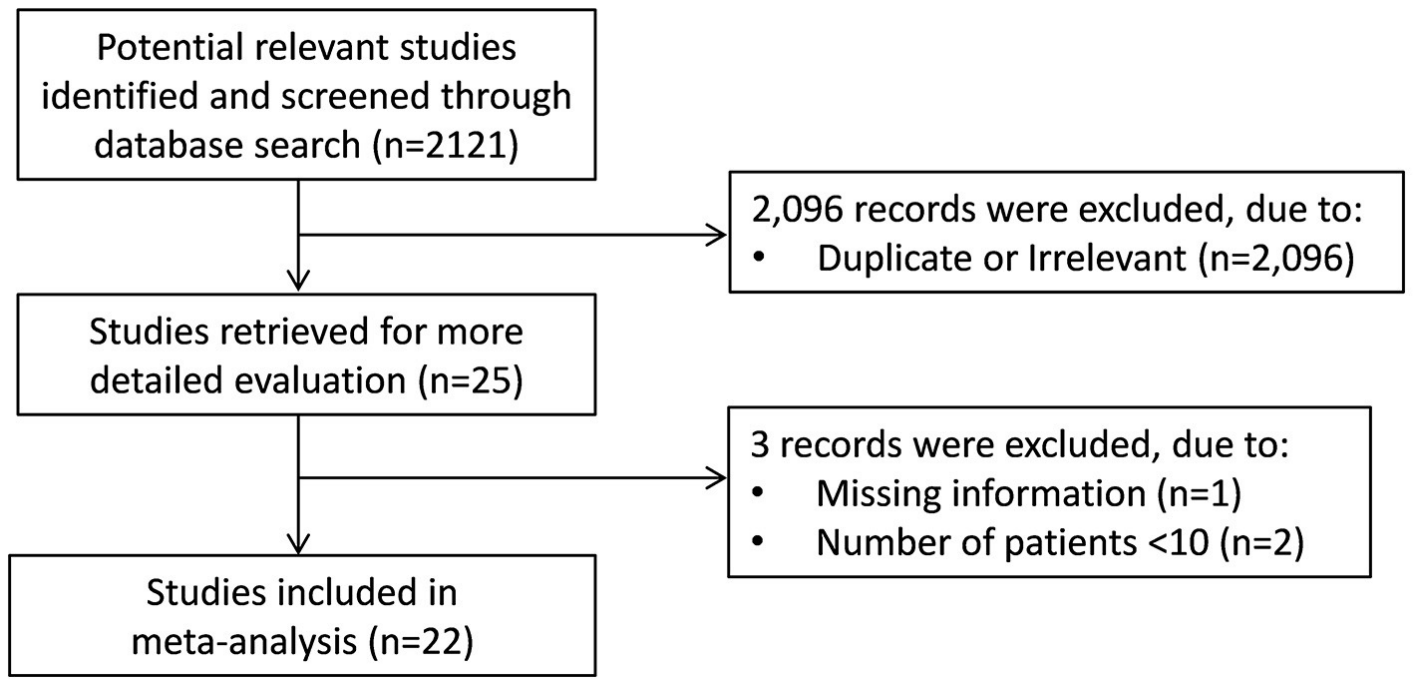
Flow chart of study selection.

A flow chart describing the screening process is presented in Figure 1. We initially identified 2,121 studies. A total of 2,096 studies were excluded after the first screening because they were either duplicates or irrelevant. After a full-text review of the remaining 25 articles, three were further excluded because they either did not provide the required information or did not meet the inclusion criteria. Thus, 22 studies were included in the meta-analysis (6, 7, 17–36).

### Total Cholesterol

**Figure 2:**
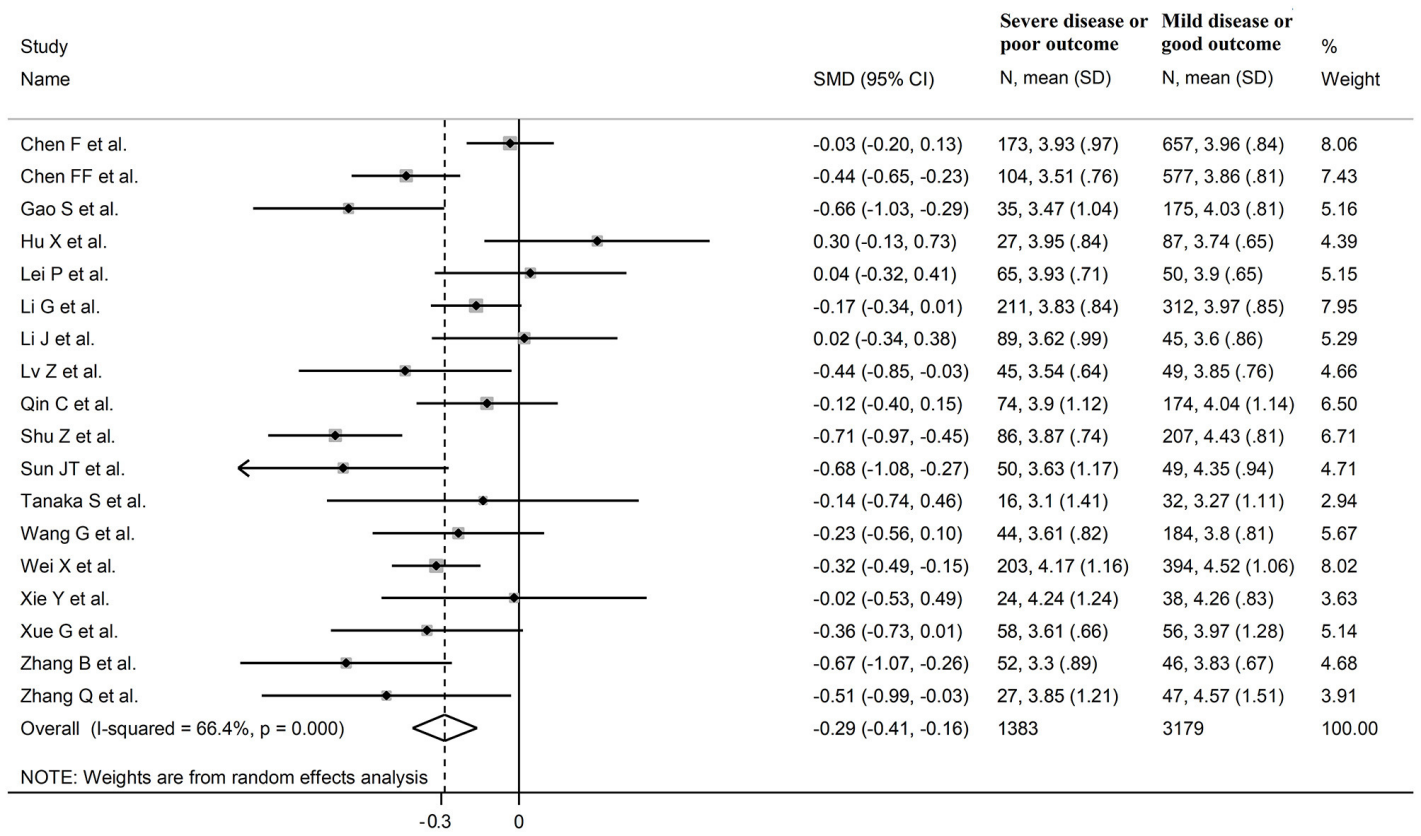
Forest plot of studies examining total cholesterol concentrations in COVID-19.

**Figure 3:**
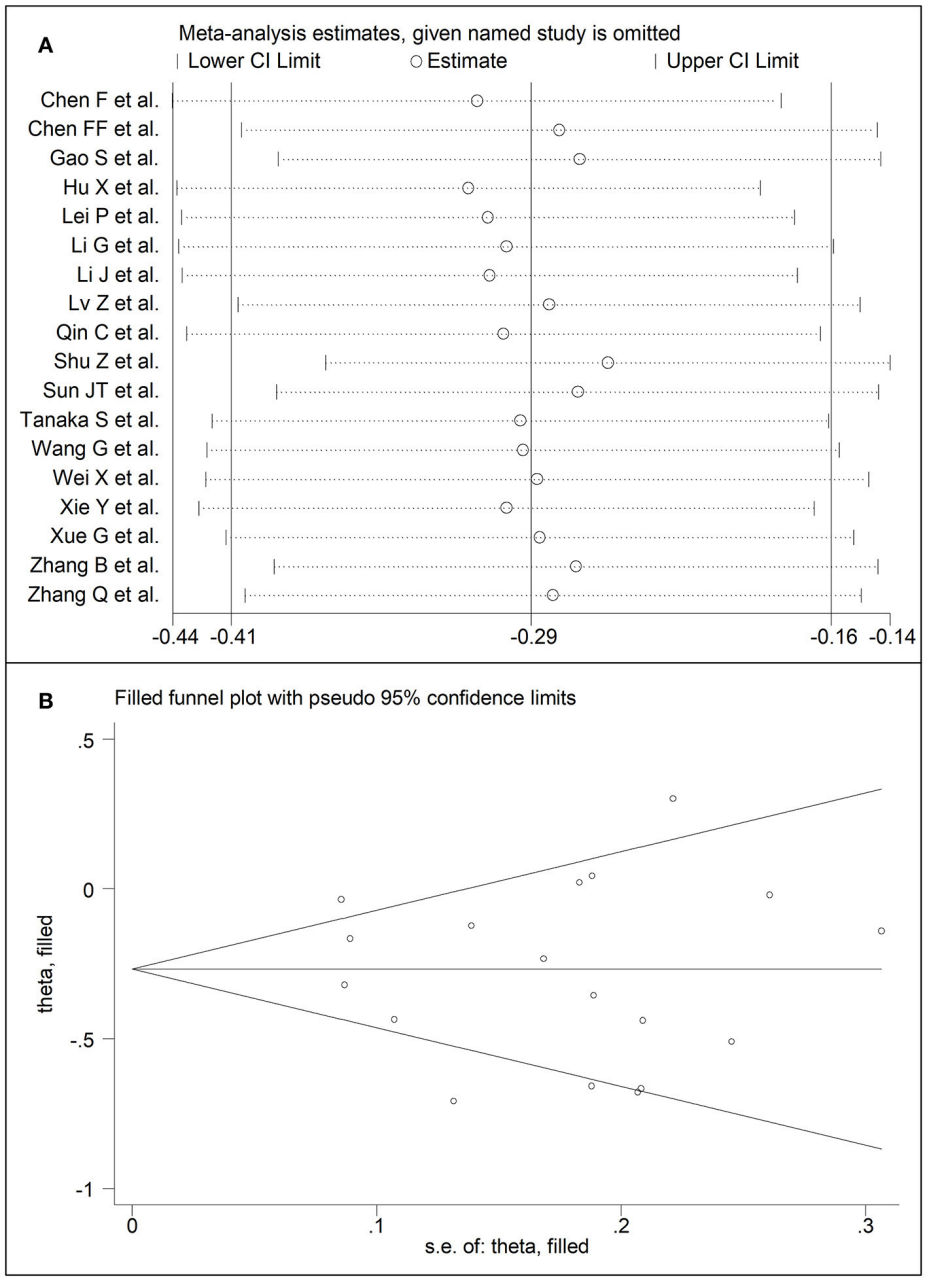
**(A)** Sensitivity analysis of the association between total cholesterol and COVID-19. The influence of individual studies on the overall standardized mean difference (SMD) is shown. The middle vertical axis indicates the overall SMD and the two vertical axes indicate the 95% confidence intervals (CIs). The hollow circles represent the pooled SMD when the remaining study is omitted from the meta-analysis. The two ends of each broken line represent the 95% CIs. **(B)** Funnel plot of studies investigating low vs. high severity or surviving vs. non-surviving status after trimming and filling. Dummy studies and genuine studies are represented by enclosed circles and free circles, respectively.

Eighteen studies assessed total cholesterol concentrations in 4,562 COVID-19 patients, 3,179 (46% males, mean age 56 years) with low severity or survivor status and 1,383 (59% males, mean age 64 years) with high severity or non-survivor status during follow up (Table 1) (6, 7, 17, 18, 20, 21, 23–26, 28–30, 32–36). All studies were conducted in China, except one, performed in France (30). Two studies were prospective (29, 30), whilst the remaining 16 were retrospective (6, 7, 17, 18, 20, 21, 23–26, 28, 32–36). Endpoints included disease severity based on current clinical guidelines in 13 studies (6, 7, 17, 21, 23, 25, 26, 28, 29, 32–34, 36), ICU admission in two (24, 35), and survival status in three (18, 20, 30). The overall standardized mean difference in total cholesterol concentrations between COVID-19 patients with low vs. high severity or survivor vs. non-survivor status is shown in Figure 2. In 15 studies, patients with high severity or non-survivor status had lower total cholesterol concentrations when compared to those with low severity or survivor status (mean difference range, −0.71 to −0.03) (6, 7, 17, 18, 20, 24, 26, 28–30, 32–36), although the difference was not statistically significant in seven studies (7, 17, 24, 30, 32–34). By contrast, in the remaining three studies (21, 23, 25), total cholesterol concentrations were lower in patients with low severity or survivor status (mean difference range, 0.02 to 0.30), even if the difference was not statistically significant. The pooled results showed that total cholesterol concentrations were significantly lower in patients with high severity or non-survivor status (SMD −0.29, 95% CI −0.41 to −0.16, *p* < 0.001), with large heterogeneity between studies (*I*^2^ = 66.4%, *p* < 0.001). In sensitivity analysis, the corresponding pooled SMD values were not altered when each study was in turn omitted (effect size ranged between −0.31 and −0.26, Figure 3A). In addition, the SMD remained significant (−0.30, 95% CI −0.43 to −0.16, *p* < 0.001) with a similar between-study variance (*I*^2^ = 62.7%, *p* < 0.001), after removing the two studies that accounted for a third of the total participants (17, 18). The Begg's (*p* = 0.83) and Egger's (*p* = 0.66) *t*-tests showed no evidence of publication bias. Accordingly, the trim-and-fill analysis did not add any study to the funnel plot (Figure 3B). In meta-regression analysis, age (*t* = −0.74, *p* = 0.47), gender (*t* = −0.21, *p* = 0.25), endpoint (*t* = −0.92, *p* = 0.37), study design (*t* = −0.78, *p* = 0.45), AST (*t* = −1.00, *p* = 0.34), ALT (*t* = −1.40, *p* = 0.19), D-dimer (*t* = −2.00, *p* = 0.10), diabetes (*t* = 0.43, *p* = 0.68), hypertension (*t* = −1.44, *p* = 0.17) and cardiovascular disease (*t* = 0.54, *p* = 0.60) were not associated with the SMD. A trend toward significance was observed with WBC (*t* = −2.01, *p* = 0.07), CRP (*t* = −1.94, *p* = 0.07), albumin (*t* = −2.31, *p* = 0.05), and creatinine (*t* = −1.94, *p* = 0.07).

**Table 1:** Characteristics of the studies in COVID-19 patients, according to disease severity or survival status, included in the meta-analysis.

		**Mild disease or survivor**							**Severe disease or non-survivor**						
**First author, country (ref)**	**NOS (stars)**	***n***	**Age (years) Mean**	**Gender (M/F)**	**TC (mmol/L) Mean ± SD**	**LDL (mmol/L) Mean ± SD**	**HDL (mmol/L) Mean ± SD**	**TG (mmol/L) Mean ± SD**	***n***	**Age (years) Mean**	**Gender (M/F)**	**TC (mmol/L) Mean ± SD**	**LDL (mmol/L) Mean ± SD**	**HDL (mmol/L) Mean ± SD**	**TG (mmol/L) Mean ± SD**
Chen et al., China (17)	7	657	62	271/386	3.96 ± 0.84	NR	NR	1.18 ± 0.62	173	65	107/66	3.93 ± 0.97	NR	NR	1.12 ± 0.68
Chen et al., China (18)	7	577	63	297/280	3.86 ± 0.81	2.41 ± 0.73	0.93 ± 0.24	1.27 ± 0.51	104	73	65/39	3.51 ± 0.76	2.12 ± 0.72	0.81 ± 0.23	1.38 ± 0.50
Deng et al., China (19)	7	53	35	24/29	NR	NR	1.09 ± 0.43	1.07 ± 0.33	12	33	12/0	NR	NR	0.83 ± 0.27	1.39 ± 0.73
Gao et al., China (20)	5	175	70	79/96	4.03 ± 0.81	NR	NR	1.23 ± 0.52	35	74	22/13	3.47 ± 1.04	NR	NR	1.37 ± 0.34
Hu et al., China (21)	6	87	46	42/45	3.74 ± 0.65	1.88 ± 0.59	1.24 ± 0.34	1.27 ± 0.60	27	62	18/9	3.95 ± 0.84	1.88 ± 0.60	1.03 ± 0.24	1.34 ± 0.42
Huang et al., China (22)	5	2,391	62	1,165/1,226	NR	2.40 ± 0.78	0.96 ± 0.26	1.33 ± 0.59	232	70	161/71	NR	1.96 ± 0.81	0.75 ± 0.27	1.65 ± 0.76
Lei et al., China (23)	5	50	65	22/28	3.90 ± 0.65	NR	NR	1.27 ± 0.47	65	69	36/29	3.93 ± 0.71	NR	NR	1.19 ± 0.50
Li et al., China (24)	7	312	49	131/181	3.97 ± 0.85	2.21 ± 0.65	1.06 ± 0.29	1.50 ± 0.89	211	62	119/92	3.83 ± 0.84	2.17 ± 0.66	0.98 ± 0.28	1.53 ± 0.71
Li et al., China (25)	6	45	50	24/21	3.60 ± 0.86	NR	NR	1.09 ± 0.58	89	64	51/38	3.62 ± 0.99	NR	NR	1.20 ± 0.60
Lv et al., China (26)	6	49	63	24/25	3.85 ± 0.76	2.50 ± 0.70	0.90 ± 0.15	1.25 ± 0.44	45	62	24/21	3.54 ± 0.64	2.13 ± 0.73	0.83 ± 0.23	1.37 ± 0.76
Petrilli et al., USA (27)	7	1,739	60	1,016/723	NR	1.84 ± 0.75	NR	NR	990	68	656/334	NR	1.47 ± 0.69	NR	NR
Qin et al., China (7)	7	174	51	91/83	4.04 ± 1.14	2.20 ± 0.91	0.86 ± 0.31	1.10 ± 0.54	74	65	39/35	3.9 ± 1.12	1.99 ± 0.75	1.03 ± 0.49	1.22 ± 0.59
Shu et al., China (28)	6	207	54	79/128	4.43 ± 0.81	2.33 ± 0.67	1.07 ± 0.22	1.60 ± 0.74	86	65	56/30	3.87 ± 0.74	2.07 ± 0.52	1.03 ± 0.22	1.43 ± 0.51
Sun et al., China (29)	7	49	52	26/23	4.35 ± 0.94	2.52 ± 0.68	1.20 ± 0.31	1.27 ± 0.73	50	71	34/16	3.63 ± 1.17	2.14 ± 0.81	0.93 ± 0.28	1.09 ± 0.68
Tanaka et al., France (30)	6	32	55	21/11	3.27 ± 1.11	1.90 ± 0.74	0.77 ± 0.44	2.13 ± 1.04	16	59	10/6	3.10 ± 1.41	1.60 ± 0.81	0.63 ± 0.30	2.23 ± 0.96
Wang et al., China (31)	5	72	44	29/43	NR	2.57 ± 0.67	1.10 ± 0.30	NR	71	65	44/27	NR	2.63 ± 0.59	0.87 ± 0.22	NR
Wang et al., China (32)	7	184	NR	NR	3.80 ± 0.81	2.66 ± 0.65	0.82 ± 0.21	1.12 ± 0.50	44	NR	NR	3.61 ± 0.82	2.58 ± 0.56	0.74 ± 0.27	1.07 ± 0.44
Wei et al., China (6)	5	394	64	189/205	4.52 ± 1.06	2.34 ± 0.54	1.30 ± 0.33	1.83 ± 0.74	203	69	116/87	4.17 ± 1.16	2.14 ± 0.63	1.24 ± 0.33	1.56 ± 0.82
Xie et al., China (33)	6	38	61	14/24	4.26 ± 0.83	2.22 ± 0.35	1.32 ± 0.34	1.33 ± 0.76	24	72	13/11	4.24 ± 1.24	2.51 ± 1.19	1.12 ± 0.43	1.34 ± 0.59
Xue et al., China (34)	7	56	61	30/26	3.97 ± 1.28	2.24 ± 0.81	1.03 ± 0.33	1.48 ± 0.56	58	64	34/24	3.61 ± 0.66	2.09 ± 0.50	0.92 ± 0.30	1.31 ± 0.52
Zhang et al., China (35)	7	46	61	24/22	3.83 ± 0.67	NR	0.97 ± 0.22	1.37 ± 0.59	52	66	34/18	3.30 ± 0.89	NR	0.77 ± 0.37	1.60 ± 0.81
Zhang et al., China (36)	6	47	61	18/29	4.57 ± 1.51	2.74 ± 0.85	1.11 ± 0.24	1.58 ± 1.02	27	72	18/9	3.85 ± 1.21	2.29 ± 1.11	0.95 ± 0.34	1.28 ± 0.65

*HDL, high-density lipoprotein; LDL, low-density lipoprotein; M/F, males/females; NOS, Newcastle-Ottawa quality assessment scale for case-control studies; NR, not reported; SD, standard deviation; TC, total cholesterol; TG, triglycerides*.

### LDL-Cholesterol

**Figure 4:**
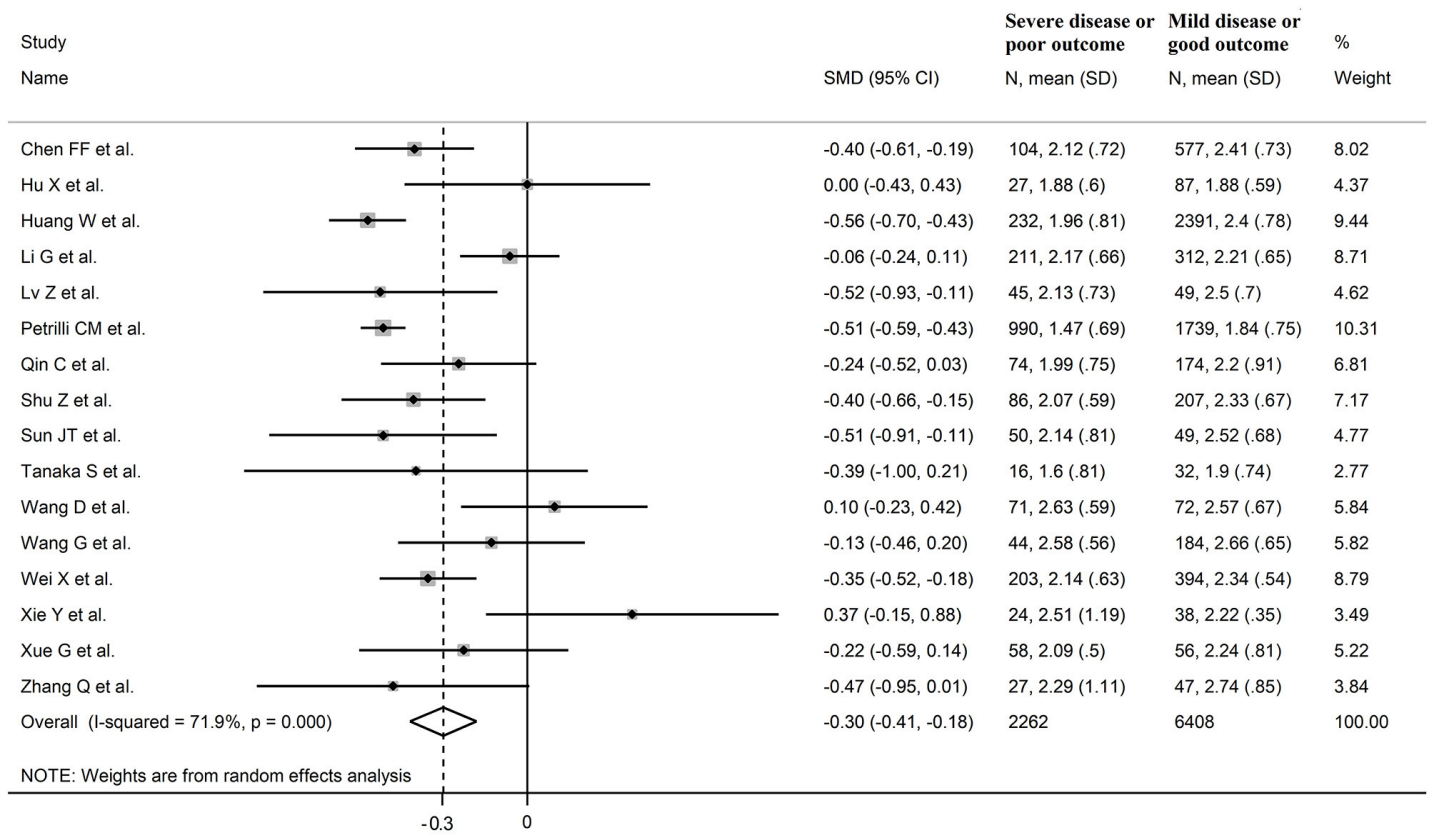
Forest plot of studies examining LDL-cholesterol concentrations in COVID-19.

**Figure 5:**
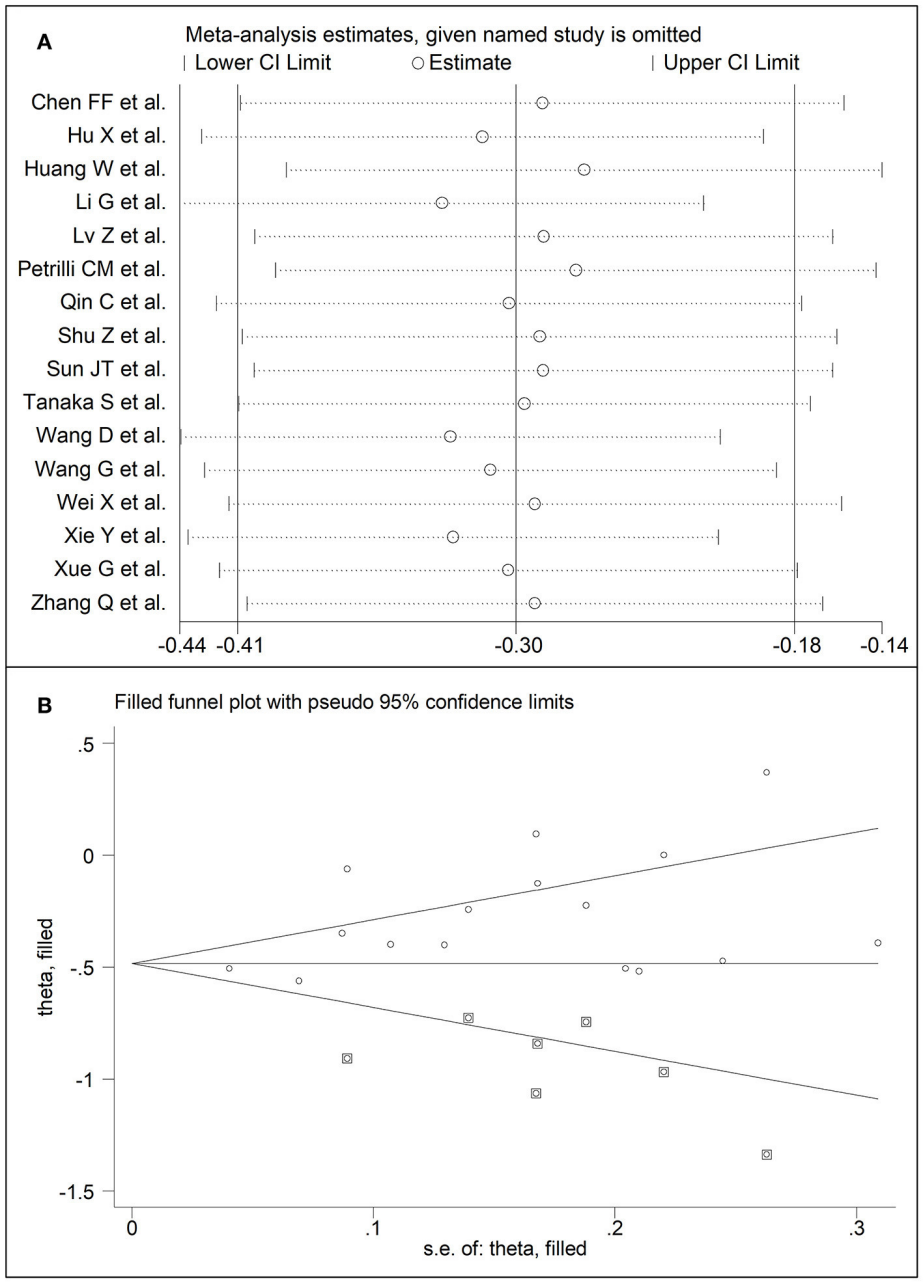
**(A)** Sensitivity analysis of the association between LDL-cholesterol and COVID-19. The influence of individual studies on the overall standardized mean difference (SMD) is shown. The middle vertical axis indicates the overall SMD and the two vertical axes indicate the 95% confidence intervals (CIs). The hollow circles represent the pooled SMD when the remaining study is omitted from the meta-analysis. The two ends of each broken line represent the 95% CIs. **(B)** Funnel plot of studies investigating low vs. high severity or surviving vs. non-surviving status after trimming and filling. Dummy studies and genuine studies are represented by enclosed circles and free circles, respectively.

Sixteen studies investigated LDL-cholesterol concentrations in 8,670 COVID-19 patients, 6,408 (51% males, mean age 60 years) with low severity or survivor status and 2,262 (63% males, mean age 68 years) with high severity or non-survivor status during follow up (Table 1) (6, 7, 18, 21, 22, 24, 26–34, 36). Fourteen studies were conducted in China (6, 7, 18, 21, 22, 24, 26, 28, 29, 31–34, 36), one in USA (27), and one in France (30). Three studies were prospective (27, 29, 30), whilst the remaining 13 were retrospective (6, 7, 18, 21, 22, 24, 26, 28, 31–34, 36). Endpoints included disease severity based on current clinical guidelines in 12 (6, 7, 21, 26–29, 31–34, 36), ICU admission in one (24), and survival status in three (18, 22, 30). The overall standardized mean difference in LDL-cholesterol concentrations between COVID-19 patients with low vs. high severity or survivor vs. non-survivor status is shown in Figure 4. In 13 studies, patients with high severity or non-survivor status had lower LDL-cholesterol concentrations when compared to those with low severity or survivor status (mean difference range, −0.56 to −0.06) (6, 7, 18, 22, 24, 26–30, 32, 34, 36), although the difference was not statistically significant in six studies (7, 24, 30, 32, 34, 36). In three studies (21, 23, 25), LDL-concentrations were non-significantly lower in patients with low severity or survivor status (mean difference range 0.10 to 0.37), whereas in one study no difference was observed between the groups (mean difference 0.00) (21). The pooled results showed that LDL-concentrations were significantly lower in patients with high severity or non-survivor status (SMD −0.30, 95% CI −0.41 to −0.18, *p* < 0.001), with large heterogeneity between studies (*I*^2^ = 71.9%, *p* < 0.001). In sensitivity analysis, the pooled SMD values were not altered when individual studies were in turn omitted (effect size ranged between −0.33 and −0.27, Figure 5A). In addition, the SMD was reduced but still significant (−0.24, 95% CI −0.35 to −0.13, *p* < 0.001), with a reduction in between-study variance (*I*^2^ = 47.9%, *p* = 0.02), after removing the two studies that accounted for 62% of all participants (22, 27). The Egger's (*p* = 0.02), but not the Begg's (*p* = 0.44), *t*-test showed the presence of publication bias. Accordingly, the trim-and-fill method identified seven potential missing studies to add on the left side of the funnel plot to ensure symmetry (Figure 5B). The adjusted SMD was further increased (−0.47, 95% CI −0.35 to −0.60, *p* < 0.001). In meta-regression analysis, age (*t* = 2.37, *p* = 0.03) and hypertension (*t* = 2.92, *p* = 0.02) were significantly and positively associated with the SMD. By contrast, non-significant relationships were observed with gender (*t* = 0.26, *p* = 0.80), endpoint (*t* = 0.11, *p* = 0.92), study design (*t* = −1.58, *p* = 0.14), country (*t* = −1.24 *p* = 0.24), AST (*t* = −0.09, *p* = 0.93), ALT (*t* = 0.23, *p* = 0.82), D-dimer (*t* = −0.67, *p* = 0.55), WBC (*t* = −0.92, *p* = 0.38), CRP (*t* = −0.54, *p* = 0.60), albumin (*t* = 0.01, *p* = 0.99), creatinine (*t* = −0.54, *p* = 0.60), diabetes (*t* = 1.64, *p* = 0.14), and cardiovascular disease (*t* = 0.67, *p* = 0.53).

### HDL-Cholesterol

**Figure 6:**
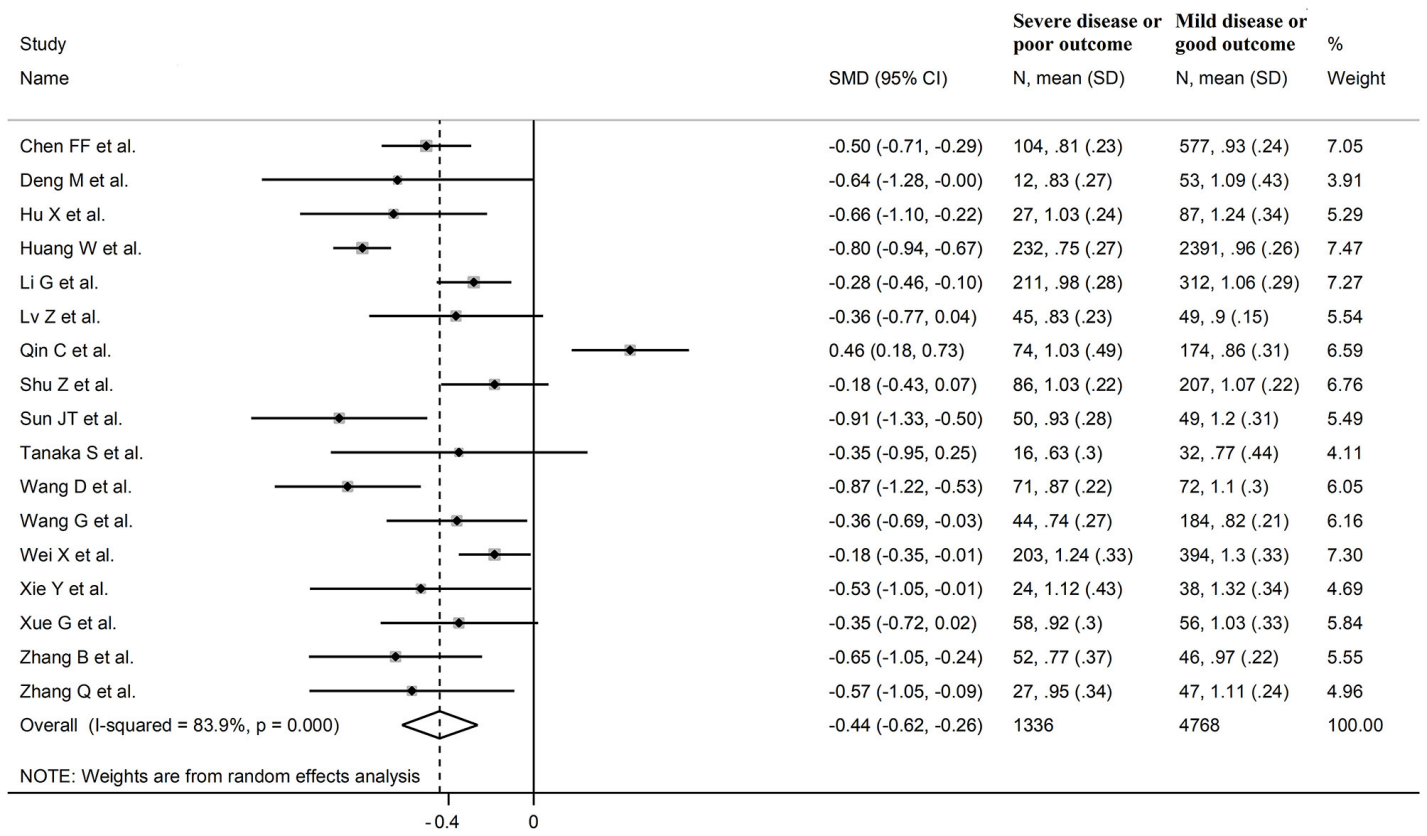
Forest plot of studies examining HDL-cholesterol concentrations in COVID-19.

**Figure 7:**
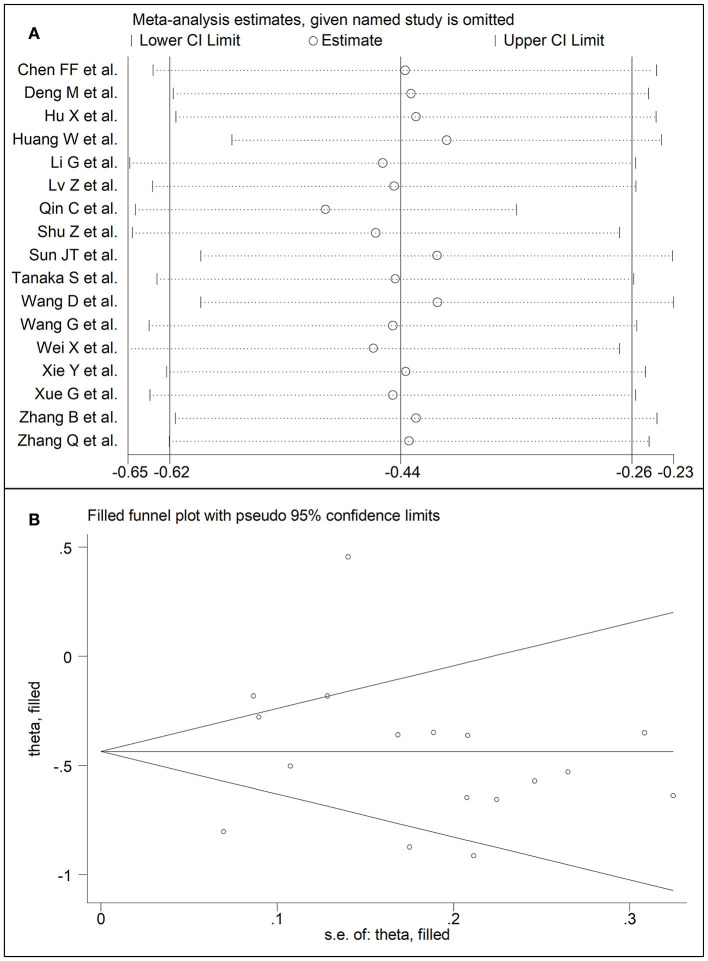
**(A)** Sensitivity analysis of the association between HDL-cholesterol and COVID-19. The influence of individual studies on the overall standardized mean difference (SMD) is shown. The middle vertical axis indicates the overall SMD and the two vertical axes indicate the 95% confidence intervals (CIs). The hollow circles represent the pooled SMD when the remaining study is omitted from the meta-analysis. The two ends of each broken line represent the 95% CIs. **(B)** Funnel plot of studies investigating low vs. high severity or surviving vs. non-surviving status after trimming and filling. Dummy studies and genuine studies are represented by enclosed circles and free circles, respectively.

Seventeen studies assessed HDL-cholesterol concentrations in 6,104 COVID-19 patients, 4,768 (48% males, mean age 60 years) with low severity or survivor status and 1,336 (62% males, mean age 67 years) with high severity or non-survivor status during follow up (Table 1) (6, 7, 18, 19, 21, 22, 24, 26, 28–36). All studies were performed in China, except one, conducted in France (30). Two studies were prospective (29, 30), whilst the remaining 15 were retrospective (6, 7, 18, 19, 21, 22, 24, 26, 28, 31–36). Endpoints included disease severity based on current clinical guidelines in 12 (6, 7, 19, 21, 26, 28, 29, 31–34, 36), ICU admission in two (24, 35), and survival status in three (18, 22, 30). The overall SMD in HDL-cholesterol concentrations between COVID-19 patients with low vs. high severity or survivor vs. non-survivor status is reported in Figure 6. In 16 studies, patients with high severity or non-survivor status had lower HDL-cholesterol concentrations than those with low severity or survivor status during follow up (mean difference range, −0.91 to −0.18) (6, 18, 19, 21, 22, 24, 26, 28–36), although the difference was not statistically significant in four (26, 28, 30, 34). In the remaining study, HDL-concentrations were significantly lower in patients with low severity or survivor status during follow up (mean difference 0.46) (7). The pooled results showed that HDL-concentrations were significantly lower in patients with high severity or non-survivor status (SMD −0.44, 95% CI −0.62 to −0.26, *p* < 0.001), with extreme heterogeneity between studies (*I*^2^ = 83.9%, *p* < 0.001). Sensitivity analysis showed that the pooled SMD values were not affected when each study was in turn omitted (effect size ranged between −0.50 and −0.41, Figure 7A). In addition, the SMD was reduced though remained significant (−0.40, 95% CI −0.58 to −0.22, *p* < 0.001), with a slight reduction in between-study variance (*I*^2^ = 76.5%, *p* < 0.001), after removing two studies accounting for 54% of all participants (18, 22). The Begg's (*p* = 0.39) and Egger's (*p* = 0.95) t-tests showed no evidence of publication bias. Accordingly, the trim-and-fill method did not add any study to the funnel plot (Figure 7B). In meta-regression analysis, age (*t* = 0.61, *p* = 0.55), gender (*t* = −1.52, *p* = 0.15), endpoint (*t* = −0.86, *p* = 0.40), study design (*t* = −0.89, *p* = 0.39), AST (*t* = −0.73, *p* = 0.48), ALT (*t* = −0.41, *p* = 0.61), D-dimer (*t* = −1.32, *p* = 0.26), WBC (*t* = −1.63, *p* = 0.13), albumin (*t* = 1.67, *p* = 0.15), diabetes (*t* = −0.57, *p* = 0.58), cardiovascular disease (*t* = −0.87, *p* = 0.41) and hypertension (*t* = −0.95, *p* = 0.37), were not significantly associated with SMD. A trend toward statistical significance was observed with CRP (*t* = −2.03, *p* = 0.07) and creatinine (*t* = −2.03, *p* = 0.07).

### Triglycerides

**Figure 8:**
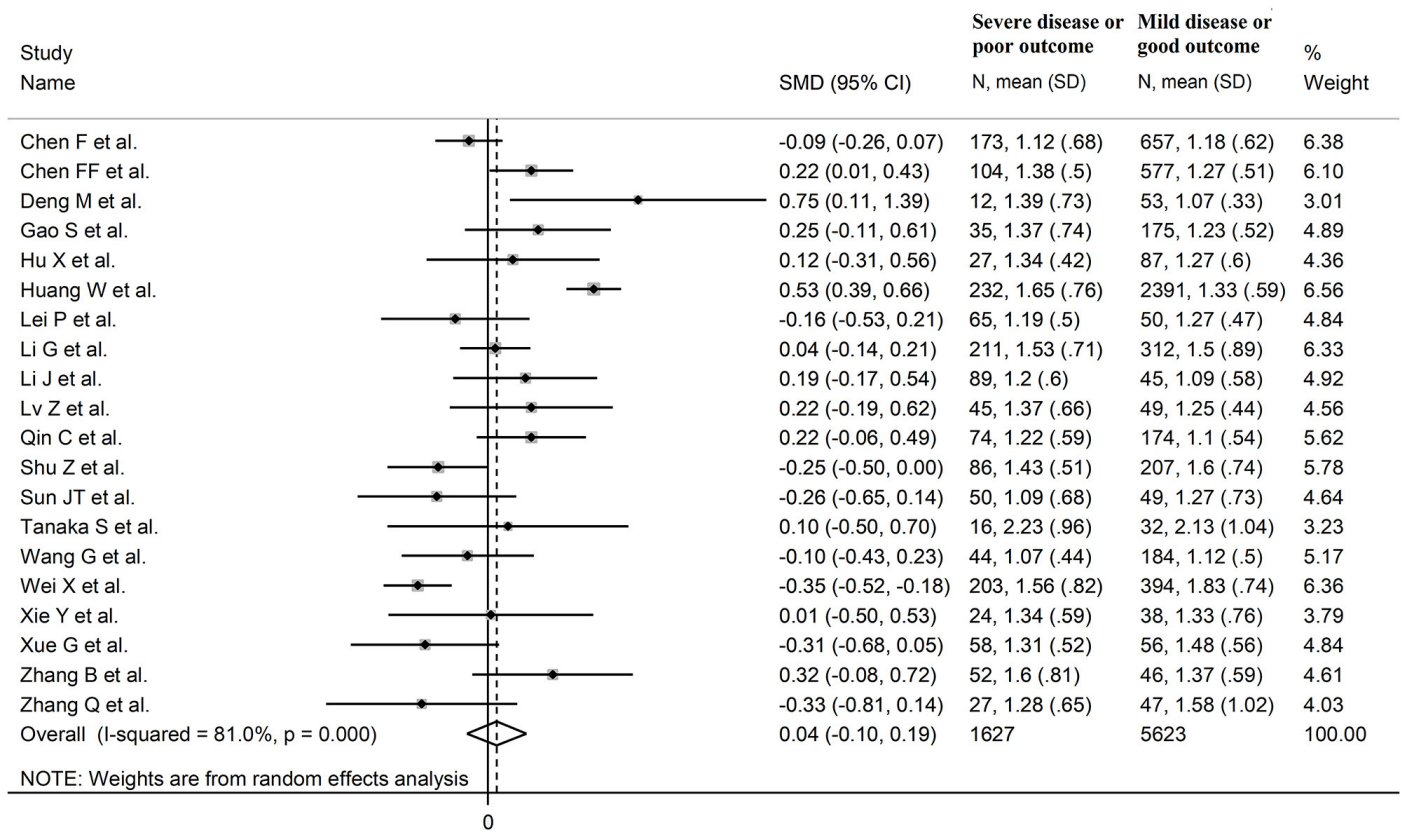
Forest plot of studies examining the concentrations of triglycerides in COVID-19.

**Figure 9:**
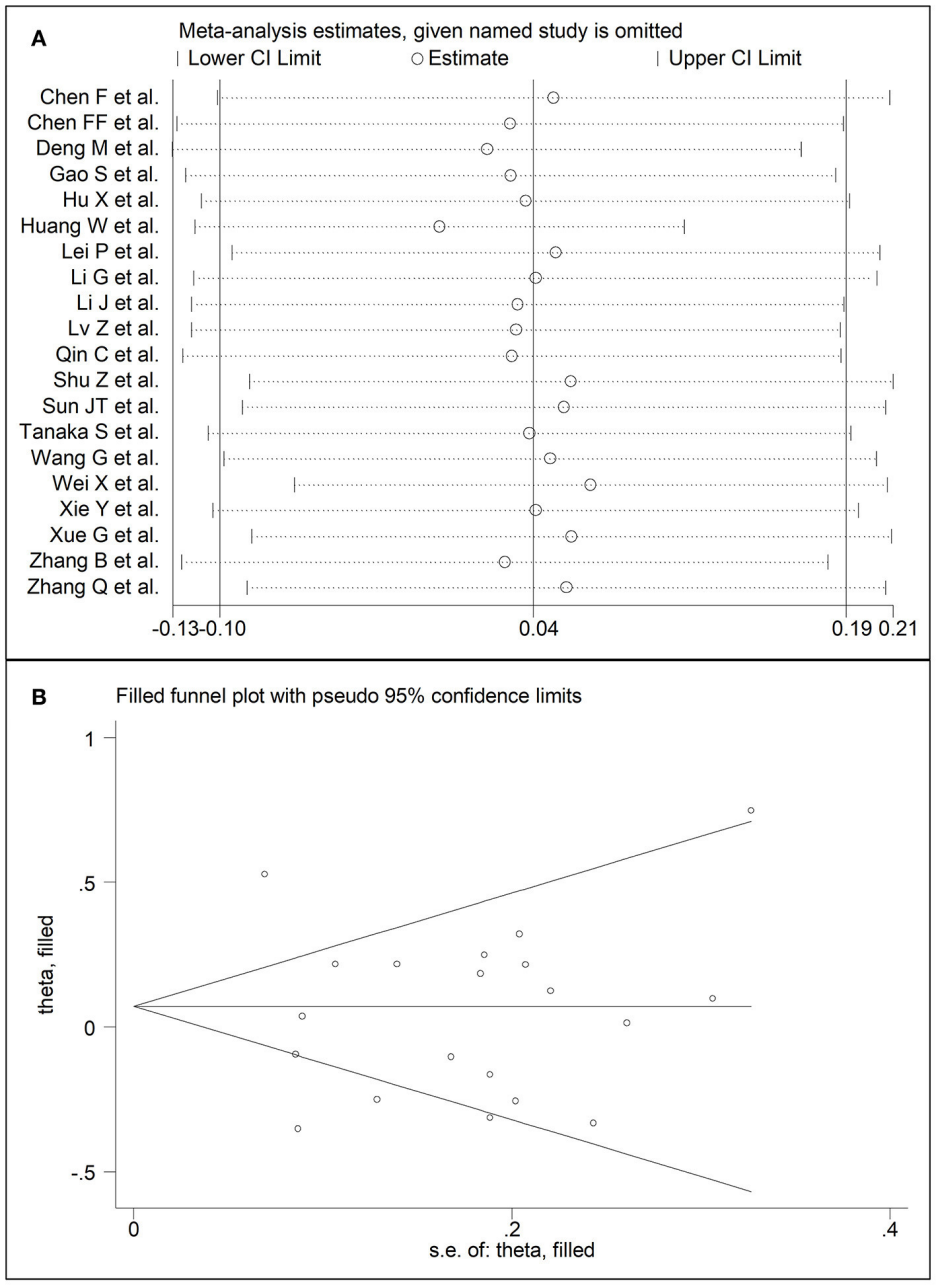
**(A)** Sensitivity analysis of the association between serum triglycerides and COVID-19. The influence of individual studies on the overall standardized mean difference (SMD) is shown. The middle vertical axis indicates the overall SMD and the two vertical axes indicate the 95% confidence intervals (CIs). The hollow circles represent the pooled SMD when the remaining study is omitted from the meta-analysis. Two ends of each broken line represent the 95% CIs. **(B)** Funnel plot of studies investigating low vs. high severity or surviving vs. non-surviving status after trimming and filling. Dummy studies and genuine studies are represented by enclosed circles and free circles, respectively.

Twenty studies reported triglyceride concentrations in 7,250 COVID-19 patients, 5,623 (47% males, mean age 60 years) with low severity or survivor status and 1,627 (61% males, mean age 67 years) with high severity or non-survivor status during follow up (Table 1) (6, 7, 17–26, 28–30, 32–36). All studies were performed in China, except one, conducted in France (30). Two studies were prospective (29, 30), whilst 18 were retrospective (6, 7, 17–26, 28, 32–36). Endpoints included disease severity based on current clinical guidelines in 14 studies (6, 7, 17, 19, 21, 23, 25, 26, 28, 29, 32–34, 36), ICU admission in two (24, 35), and survival status in four (18, 20, 22, 30). The overall SMD in triglyceride concentrations between patients with low vs. high severity or survivor vs. non-survivor status is reported in Figure 8. In eight studies, patients with high severity or non-survivor status had lower triglyceride concentrations when compared to those with low severity or survivor status during follow up (mean difference range, −0.35 to −0.09) (6, 17, 23, 28, 29, 32, 34, 36), although the difference was statistically significant only in one study (6). In 12 studies (7, 18–22, 24–27, 30, 33, 35), triglyceride concentrations were lower in patients with low severity or survivor status (mean difference range, 0.04 to 0.75), although the difference was statistically significant only in three studies (18, 19, 22). The pooled results showed that triglyceride concentrations were similar in the two groups (SMD 0.04, 95% CI −0.10 to −0.19, *p* = 0.57), with extreme heterogeneity between studies (*I*^2^ = 81.0%, *p* < 0.001). Sensitivity analysis showed that the pooled SMD values were not altered when each study was in turn removed (effect size ranged between 0.00 and 0.07, Figure 9A). In addition, the SMD remained non-significant (0.10, 95% CI −0.12 to 0.14, *p* = 0.88), but with a reduction in between-study variance (*I*^2^ = 62.8%, *p* < 0.001), after removing two studies that accounted for 48% of all participants (17, 22). The Begg's (*p* = 0.67) and Egger's (*p* = 0.58) *t*-tests showed no evidence of publication bias. Accordingly, the trim-and-fill method did not add any study to the funnel plot (Figure 9B). Meta-regression analysis showed that endpoint (*t* = 3.29, *p* = 0.004) and AST (*t* =2.65, *p* = 0.02) were significantly associated to the effect size, with a trend toward significance for albumin (*t* = 2.08, *p* = 0.07). By contrast, age (*t* = −0.50, *p* = 0.62), gender (*t* = 0.44, *p* = 0.66), study design (*t* = −0.68, *p* = 0.51), ALT (*t* = 0.94, *p* = 0.36), D-dimer (*t* = −0.44, *p* = 0.65), WBC (*t* = 1.72, *p* = 0.11), CRP (*t* = −0.15, *p* = 0.88), creatinine (*t* = −0.15, *p* = 0.88), diabetes (*t* = 0.38, *p* = 0.71), cardiovascular disease (*t* = −1.06, *p* = 0.32) and hypertension (*t* = −0.36, *p* = 0.73) were not significantly associated with the SMD.

## Discussion

In our systematic review and meta-analysis, the serum/plasma concentrations of total cholesterol, LDL-cholesterol, and HDL-cholesterol were significantly lower in COVID-19 patients with more severe disease, ascertained clinically or with documented transfer to ICU, and in those who did not survive during follow up when compared to patients with milder forms of the disease or who survived during follow up. By contrast, no significant associations were observed between triglyceride concentrations, COVID-19 severity, and mortality. The observed SMD values for total cholesterol, LDL-cholesterol, and HDL-cholesterol, −0.29, −0.30, and −0.44, respectively, indicate an effect size that is likely to be of biological and/or clinical relevance (37). The heterogeneity between studies was generally large-to-extreme however in sensitivity analysis the effect size of different lipid fractions was not significantly affected when each study was in turn removed. Further analyses based on the Begg's and Egger's *t*-tests did not show evidence of significant publication bias, except for LDL-cholesterol. With this lipid fraction, the trim-and-fill method identified seven potential missing studies to add on the left side of the funnel plot to ensure symmetry. In meta-regression analysis, performed to identify specific study, clinical and demographic factors potentially associated with the SMD, only age and hypertension were significantly associated with the SMD for LDL whereas the type of study endpoint and AST values were significantly associated with the SMD for triglycerides.

The exact mechanisms responsible for the lower plasma/serum concentrations of total cholesterol, LDL-cholesterol, and HDL-cholesterol in patients with COVID-19, particularly in those with the more severe forms of the disease, are unclear. A reduction in cholesterol fractions has been previously reported with other viral agents, particularly the Human Immunodeficiency Virus-1 (HIV-1). In patients with HIV-1 infection, a significant reduction in plasma/serum concentrations of HDL-cholesterol has been linked with the impaired function of the ATP-binding cassette transporter A1-dependent cholesterol efflux in macrophages and the activation of endothelial lipase and phospholipase A2, mediated by a pro-inflammatory state (38–40). While alterations in these transporters and enzymes have not been specifically reported in patients with COVID-19, other mechanisms may be involved in this group. For example, it has been speculated that the increased utilization of cholesterol for the synthesis of pulmonary surfactant, as a response to viral infection and replication in the lungs, and/or a poor nutritional status associated with reduced liver synthetic capacity, might account, at least in part, for the observed hypocholesterolemia in severe COVID-19 (7). The latter hypothesis is supported by the results of a recent systematic review and meta-analysis which reported that lower concentrations of serum prealbumin, a combined marker of malnutrition and inflammation, are also significantly associated with higher COVID-19 severity and adverse clinical outcomes (41). It is also possible that the non-specific presence of sepsis, similar to other bacterial and viral infections, can lead to the observed alterations in lipid profile through the activation of specific pro-inflammatory cytokines and/or the increased expression of the scavenger receptor class B type 1 (6, 42). While previous studies have reported an inverse association between CRP and HDL-cholesterol concentrations in COVID-19, the results of our meta-regression analysis showed a non-significant trend for an association between the SMD for HDL-cholesterol and CRP (*t* = −2.03, *p* = 0.07). Further experimental and human studies are required to clarify whether the association between an excessive inflammatory state and HDL-cholesterol, and other lipid fractions, in patients with severe COVID-19 is mediated by non-specific inflammatory markers or individual cytokines. The significant differences observed in total-cholesterol, HDL-cholesterol, and LDL-cholesterol concentrations between COVID-19 patients with different severity and clinical outcomes could also be theoretically amplified, at least in part, by the different pre-admission and/or hospital use of cholesterol lowering agents, particularly statins, in these subgroups. However, a systematic review and meta-analysis of European and North American studies in 2,398 patients with COVID-19 has recently reported that the use of statins was associated with a significantly reduced risk of disease progression or mortality (odds ratio, OR, 0.59, 95% CI 0.35 to 0.99, *p* = 0.02). This trend persisted after excluding studies where statins were commenced during hospital admission (OR 0.51, 95% CI 0.41 to 0.64, *p*-value not reported) (43). Pending confirmation in other ethnic groups, for example, Chinese patients, these results suggest the presence of a complex interplay between lipid profile on admission, pre-hospital and in-hospital statin use, disease severity and mortality in COVID-19. The limited information available on the use of statins in the studies identified in our systematic review prevented the conduct of meta-regression analysis to investigate associations between statin use and the SMD of various lipid fractions. This important issue requires further research as the results of *in vitro* experiments support the presence of anti-viral effects of statins against SARS-CoV-2 (44).

The large-to-extreme between-study heterogeneity represents a potential limitation in our study. However, there was no evidence of publication bias, barring studies reporting LDL-cholesterol concentrations, and the overall effect size was not significantly influenced in sensitivity analyses. The lack of significant associations between study, clinical, and demographic characteristics and the SMD, barring the associations between age and hypertension and the SMD for LDL and between the type of study design and AST and the SMD for triglycerides previously described, suggest that other unreported factors, for example, statin use and/or issues with standardization of the analytical methods for the measurement of different lipid fractions (45), might contribute to the observed heterogeneity. An additional limitation in our study was the lack of information provided in most studies regarding the exact timing of the blood collection for lipid profile, for example, on the day of admission or thereafter.

In conclusion, our systematic review and meta-analysis has shown that lower plasma/serum concentrations of total cholesterol, LDL-cholesterol, and HDL-cholesterol, but not triglycerides, are significantly associated with more severe disease and increased mortality in patients with COVID-19. While the assessment of lipid profile, with or without other patient characteristics, might assist with risk stratification, additional prospective studies are required to investigate the relationship between various cholesterol fractions and statin use, the temporal variations in lipid concentrations, and the clinical impact of these variables in this patient group.

## Data Availability Statement

The raw data supporting the conclusions of this article will be made available by the authors, without undue reservation.

## Author Contributions

AZ and AM: initial idea. AZ, PP, and PS: data collection and analysis. AZ, PP, AF, PS, CC, and AM: data interpretation and writing—review and editing. AM: writing—first draft. All authors contributed to the article and approved the submitted version.

## Conflict of Interest

The authors declare that the research was conducted in the absence of any commercial or financial relationships that could be construed as a potential conflict of interest.

## Publisher's Note

All claims expressed in this article are solely those of the authors and do not necessarily represent those of their affiliated organizations, or those of the publisher, the editors and the reviewers. Any product that may be evaluated in this article, or claim that may be made by its manufacturer, is not guaranteed or endorsed by the publisher.
